# Prediction of unerupted canines and premolars widths in an Emirati population: development and validation of regression and machine learning models

**DOI:** 10.1038/s41598-026-54292-8

**Published:** 2026-05-21

**Authors:** Nour Alnusairat, Ali I. Ibrahim, Hasna Alsaeed, Widad Nsairat, Amar H. Khamis, Nameer Al-Taai

**Affiliations:** 1 Hamdan Bin Mohammed College of Dental Medicine, Mohammed Bin Rashid University of Medicine and Health Sciences, Dubai, UAE; 2 Faculty of Dentistry, Oral & Craniofacial Sciences, Centre for Oral, Clinical and Translational Sciences, King’s College London, London, UK; 3 Department of Orthodontics, College of Dentistry, University of Baghdad, Baghdad, Iraq; 4 Orthodontic Unit, Oral Health Department, Dubai Health, Dubai, UAE; 5 Private Practice, Abu Dhabi, UAE; 6 Orthodontics, Department of Odontology, Umeå University, Umeå, Sweden

**Keywords:** Mixed dentition analysis, Tanaka–Johnston equation, Unerupted canine–premolar prediction, Machine learning in orthodontics, Anatomy, Diseases, Health care, Medical research

## Abstract

This study aimed to develop a more accurate model for predicting the widths of unerupted canines and premolars in Emirati children, using deep learning and machine learning techniques. Dental models of 380 Emirati individuals aged 15–30 years were collected. The mesiodistal widths of permanent teeth were measured with a standardized orthodontic digital caliper. Regression models were developed using linear regression, Support Vector Regression (SVR; machine learning), and Artificial Neural Networks (ANN; deep learning). The widths of mandibular lateral incisors, central incisors, and the summed width of mandibular incisors were used as predictors. A two-tailed paired t-test was used to assess differences between measured and predicted values. Model performance was evaluated using pass rate (defined as predictions within ± 1 mm of measured values), mean absolute error (MAE), root mean square error (RMSE), and coefficient of determination (R^2^). The dataset was randomly divided into training (70%), validation (20%), and test (10%) sets. A statistically significant difference (*P* < 0.001) was found between the values predicted by the Tanaka–Johnston equations and the measured values. In contrast, no significant differences (*P* > 0.05) were observed between the measured values and those predicted by newly derived models. The highest average pass rate (78.5%, MAE 0.66) was achieved with linear regression using one predictor (summed width of the mandibular incisors). The Tanaka–Johnston method showed limited validity in the Emirati population. Population-specific regression equations significantly improved prediction accuracy, while machine-learning approaches enhanced model stability without outperforming well-calibrated linear regression models, supporting the use of simple, interpretable models for clinically reliable mixed-dentition space analysis.

## Introduction

The mixed dentition period is characterized by dynamic dental changes that directly influence occlusal development^1^. Early diagnosis at this stage enables clinicians to anticipate future crowding or spacing and to implement timely interceptive strategies to optimize long-term occlusal outcomes^2^. Among the most common challenges during mixed dentition is dental crowding, which may negatively affect oral hygiene, periodontal health, facial aesthetics, and psychosocial well-being if left untreated^3,4^.

Accurate prediction of the mesiodistal widths of unerupted permanent canines and premolars is essential for space analysis in mixed dentition. Mixed dentition space analysis (MDSA) evaluates whether there is sufficient arch length to accommodate the unerupted teeth by comparing available space with the predicted tooth size^5^. This analysis directly contributes to important treatment decisions, including space maintenance, space regaining, serial extraction, eruption guidance, or monitoring dental development^2^. Given their impact on extraction decisions and long-term treatment outcomes, the accuracy and reliability of MDSA methods are of significant clinical importance.

Maxillary canines are essential in orthodontic diagnosis due to their role in occlusion and esthetics, and their late eruption makes them prone to disturbances such as impaction. Accurate prediction of the mesiodistal widths of unerupted canines and premolars is therefore essential for space analysis and treatment planning. Inaccurate estimation can lead to clinical errors, with overestimation resulting in unnecessary extractions and underestimation increasing the risk of crowding, underscoring the need for reliable prediction methods.

Several methods have been proposed to estimate the mesiodistal widths of unerupted teeth. Radiographic methods, first described by Bull^6^, involve direct measurement of unerupted teeth from radiographs after correction for magnification. Although these techniques may provide relatively accurate estimates, their clinical utility is limited due to radiation exposure, technical sensitivity, cost, and dependence on operator skill^7,8^. Consequently, non-radiographic methods have gained widespread acceptance. Among these, Moyers’ probability tables and the Tanaka–Johnston regression equations remain the most widely used methods due to their simplicity and clinical applicability^5,9^.

Despite their popularity, conventional prediction methods exhibit well-documented limitations. Both Moyers and Tanaka–Johnston methods were developed using data from predominantly Caucasian populations and are based on averaged linear relationships between erupted and unerupted teeth^5,9^. Several validation studies have shown that these methods often underestimate or overestimate tooth width when applied to populations with different ethnic backgrounds, reflecting significant ethnic and sexual dimorphism in tooth widths^10–12^. Inaccurate space analysis may lead to inappropriate extraction decisions, affecting occlusal stability and facial soft-tissue outcomes^10^. These results emphasize the need for population-specific prediction models.

In light of recent technological developments, artificial intelligence (AI), machine learning (ML), and deep learning (DL) methods have emerged as promising tools for orthodontic prediction tasks^13,14^. Unlike traditional linear regression models, ML and DL techniques can capture complex, non-linear relationships among multiple variables and thus may provide superior predictive performance in biologically variable systems. Studies applying neural network models for MDSA showed accuracy comparable to or exceeding that of traditional methods, highlighting the potential of AI-powered models to generate population-specific predictions while reducing operator dependence^13,14^.

In recent years, artificial intelligence and machine learning have been increasingly applied in orthodontics to improve diagnostic accuracy and predictive modeling. These approaches enhance the prediction of tooth size and treatment outcomes by identifying complex patterns in clinical data and may complement traditional regression methods^15,16^.

Despite growing interest in AI applications in orthodontics, there is still a lack of data evaluating machine learning and deep learning models for MDSA in Middle Eastern populations, particularly among Emirati children. Considering the documented ethnic differences in tooth width, relying on prediction models developed for other populations may compromise diagnostic accuracy in this group. Therefore, the present study aimed to develop and evaluate population-specific regression and machine-learning models for predicting the mesiodistal widths of unerupted permanent canines and premolars in an Emirati population, with the objective of improving the accuracy of mixed-dentition space analysis and clinical decision-making. The null hypothesis was that there would be no significant difference between measured values and those predicted by the Tanaka–Johnston method in the Emirati population. In addition, it was hypothesized that machine-learning models would not demonstrate superior predictive performance compared with conventional regression approaches.

## Methods

### Study design and ethical considerations

This retrospective cross-sectional analytical study was conducted at the Orthodontics Department of Hamdan Bin Mohammed College of Dental Medicine. The study involved the use of pre-orthodontic study models obtained from patient records. Written informed consent for the use of anonymized clinical records, including dental models, for research purposes was obtained from all participants and/or their guardians prior to data collection. Ethical approval was obtained from the Dubai Scientific Research Ethics Committee–Dubai Health (IRB approval number: 2024-117), and all procedures adhered to institutional ethical guidelines. Participant confidentiality was ensured through anonymized coding and secure storage of data on a password-protected university OneDrive server in accordance with institutional data-protection policies.

### Sample size calculation

Sample size was determined based on an estimate of clinical prediction accuracy, defined as the proportion of predicted values within ± 1.0 mm of the measured combined mesiodistal widths. Using the reported prediction accuracy proportion (*P* = 0.495)^13^, a precision-based calculation for a single proportion at the 95% confidence level with ± 5% absolute precision yielded a minimum sample size of approximately 384 subjects. A sample of 380 individuals provided comparable precision (≈ ± 5%) and was therefore considered sufficient for regression modeling and machine learning model development and validation.

Although the sample size was calculated using a clinically relevant precision approach, it also exceeded commonly recommended thresholds for regression modeling, given the number of predictors. In addition, repeated training–validation–test splits were used to enhance model stability and reduce overfitting.

### Study sample and data collection

Pretreatment dental models of 380 Emirati individuals (220 males and 160 females), aged 15–30 years, with at least one previous generation of Emirati ancestry, were collected from orthodontic departments of primary health centers in Dubai and Abu Dhabi. Participants were included if they were Emirati nationals with at least one parental Emirati generation, as determined from clinical records and self-reported demographic information collected at registration.

The records were collected from multiple centers operating under standardized clinical protocols within the same healthcare system, thereby ensuring consistent data collection.

Although the study aimed to develop prediction models applicable to children in the mixed dentition stage, measurements were obtained from individuals aged 15–30 years to ensure complete eruption of permanent teeth and accurate assessment of mesiodistal tooth dimensions, as is standard practice in mixed dentition analysis studies. Dental models were required to demonstrate fully erupted permanent dentition from first molar to first molar in both arches, with all teeth accessible for accurate mesiodistal measurements. Only models free of interproximal caries or restorations, abnormal tooth morphology or size, fractures, clinically significant tooth wear, and other dental anomalies were included. Subjects with a history of previous orthodontic treatment, missing or supernumerary teeth, proximal restorations, dental caries, attrition, or any condition that could compromise accurate mesiodistal measurement were excluded. These criteria were established in accordance with previously published mixed dentition prediction studies^12,17,18^.

### Measurement protocol

**Fig. 1:**
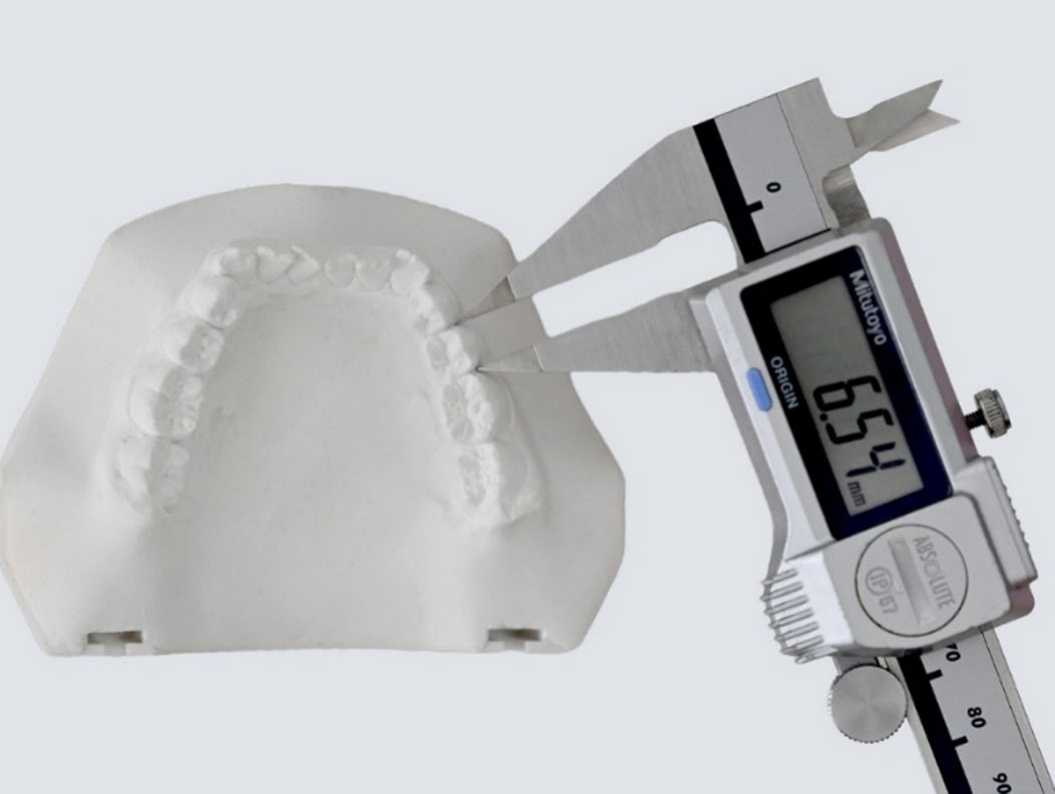
Measurement of the greatest mesiodistal crown width between anatomical contact points, with the caliper positioned parallel to the occlusal plane and perpendicular to the long axis of the tooth.

Mesiodistal crown widths of maxillary and mandibular canines, premolars, and mandibular incisors were measured by a single calibrated examiner (NAN) using a digital caliper (Mitutoyo, Japan; range 0–150 mm; accuracy 0.01 mm) (Fig. 1). Measurements followed the standardized protocol described by Moorrees and Reed (1964) and Tanaka and Johnston (1974)^9,19^, recording the greatest mesiodistal width between anatomical contact points with the caliper oriented parallel to the occlusal plane and perpendicular to the long axis of the tooth (Fig. 1). Digital calipers have been shown to provide highly accurate measurements of dental casts^20–23^.

Blinding of the examiner was not feasible during measurement; however, the examiner was not involved in the statistical analysis or model development, and standardized measurement protocols and high intra- and inter-examiner reliability were used to minimize potential measurement bias.

Because no statistically significant differences between right and left antimeres have been reported in previous studies^18,21^, corresponding canine and premolar widths were averaged before analysis.

The clinical error threshold for prediction models was defined as an absolute difference ≤ 1 mm between predicted and measured values, consistent with orthodontic literature^13,14^.

### Error of method

To evaluate intraobserver reliability, 10 models (160 tooth measurements) were randomly selected and remeasured after a 4-week interval by the same examiner (NAN). Interobserver reliability was assessed by a second examiner (NAT), who independently measured the same models after a 2-month interval. Intraclass correlation coefficients (ICCs) with 95% confidence intervals and Bland–Altman analysis were used to evaluate measurement reliability.

### Tanaka–Johnston prediction method

Predicted combined mesiodistal widths of canines and premolars were calculated using the Tanaka–Johnston Eqs. ^9^. For the maxillary arch, 11.0 mm was added to one-half of the combined mesiodistal width of the four mandibular incisors. For the mandibular arch, 10.5 mm was added to one-half of the combined mandibular incisor width. Predicted values were compared with measured values using paired t-tests and Bland–Altman analysis.

### Derivation of new prediction equations

New regression equations were developed separately for each arch using ordinary least-squares linear regression. The dependent variable was the measured combined mesiodistal width of canines and premolars, whereas predictor variables included mandibular central incisor width, mandibular lateral incisor width, and combined mandibular incisor width. Regression equations were expressed as:

$${\mathrm{Y}} = {\mathrm{A}} + {\mathrm{B}}\left( {\mathrm{X}} \right)$$

where Y represents predicted canine–premolar width per quadrant, X represents mandibular incisor width, and A and B are regression constants.

### Machine learning and regression models

Prediction models were developed using linear regression, support vector regression (SVR), and artificial neural networks (ANN) implemented in MATLAB (MathWorks, USA). Predictor variables included mandibular central incisor width, lateral incisor width, and combined mandibular incisor width.

The dataset was randomly divided into training (70%), validation (20%), and test (10%) subsets. Linear regression models were developed using the *fitlm* function, including models with and without intercept terms. The SVR model was implemented using a linear kernel with feature standardization. A linear kernel was chosen due to the single-predictor structure and the expected linear relationship between variables, thereby maintaining model interpretability and clinical relevance.

ANN models were implemented employing the *fitrnet* function. The ANN model was implemented as a feedforward neural network with a single hidden layer of 10 neurons. The hidden layer used a tangent sigmoid activation function, and the output layer used a linear activation function. Training was conducted using the Levenberg–Marquardt algorithm with mean squared error as the loss function. Given the limited number of predictors and the relatively simple structure of the dataset, model complexity was intentionally restrained to reduce the risk of overfitting. Extensive hyperparameter optimization was not performed, as more complex model configurations or nonlinear kernels were not expected to provide meaningful advantages and would have reduced model interpretability.

To enhance robustness and minimize overfitting, model training and evaluation were conducted using repeated random data splits (70/20/10) across multiple iterations, and performance metrics were averaged across runs rather than depending on a single partition. The 70/20/10 split was selected to balance training stability and independent evaluation. Given the dataset size and the model’s simplicity, allocating a larger test set would have reduced the available training data without substantially improving evaluation reliability.

**Fig. 2:**
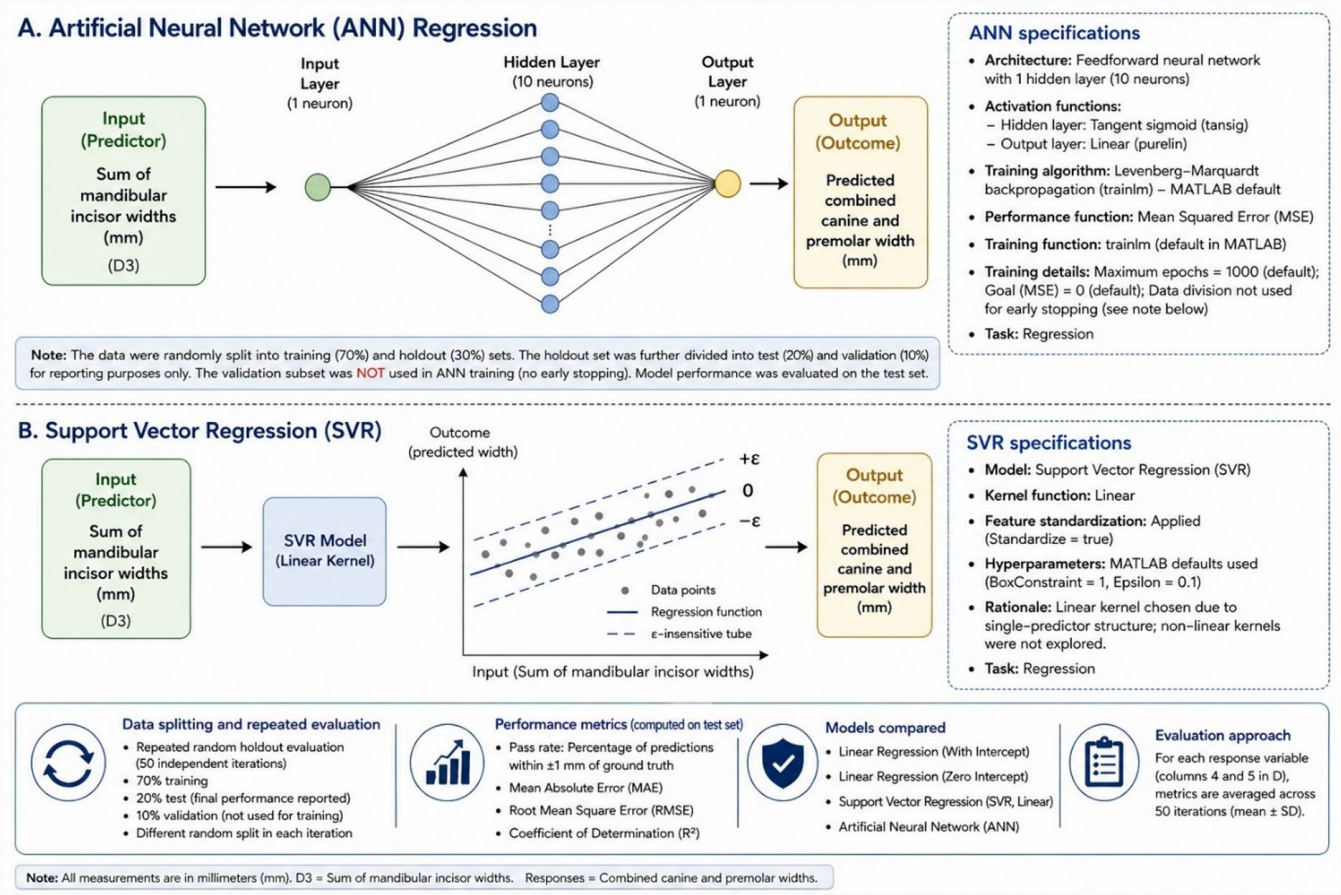
Schematic representation of the machine-learning models used for predicting the combined mesiodistal widths of unerupted canines and premolars. The input variable was the sum of mandibular incisor widths. Models included (**A**) artificial neural networks (ANN) and (**B**) support vector regression (SVR) with a linear kernel. Performance was evaluated using repeated training–validation–test splits and standard metrics (pass rate, MAE, RMSE, and R^2^).

Model performance was evaluated using pass rate (defined as predictions within ± 1 mm of measured values), mean absolute error (MAE), root mean square error (RMSE), and coefficient of determination (R^2^). A schematic representation of the machine-learning models used in this study is shown in Fig. 2.

### Model validation and pass rate analysis

Model performance was assessed by comparing predicted and measured values using paired t-tests and Bland–Altman analysis to estimate agreement and systematic bias. Clinical prediction accuracy was quantified using the pass rate, described as the proportion of predictions within ± 1 mm of the measured values, which represents a clinically acceptable threshold.

### Statistical analysis

Data normality was assessed using the Anderson–Darling, Shapiro–Wilk, and D’Agostino–Pearson tests. Accordingly, parametric or nonparametric statistical tests were applied as appropriate, with paired t-tests for normally distributed data and Wilcoxon signed-rank tests otherwise. Differences between predicted and measured values were evaluated using paired t-tests or Wilcoxon signed-rank tests as appropriate (GraphPad Prism 8.4.2; α = 0.05). Agreement between predicted and measured values was assessed using Bland–Altman analysis.

## Results

Intra-observer and inter-observer reliability were excellent (ICC > 0.90).

**Fig. 3:**
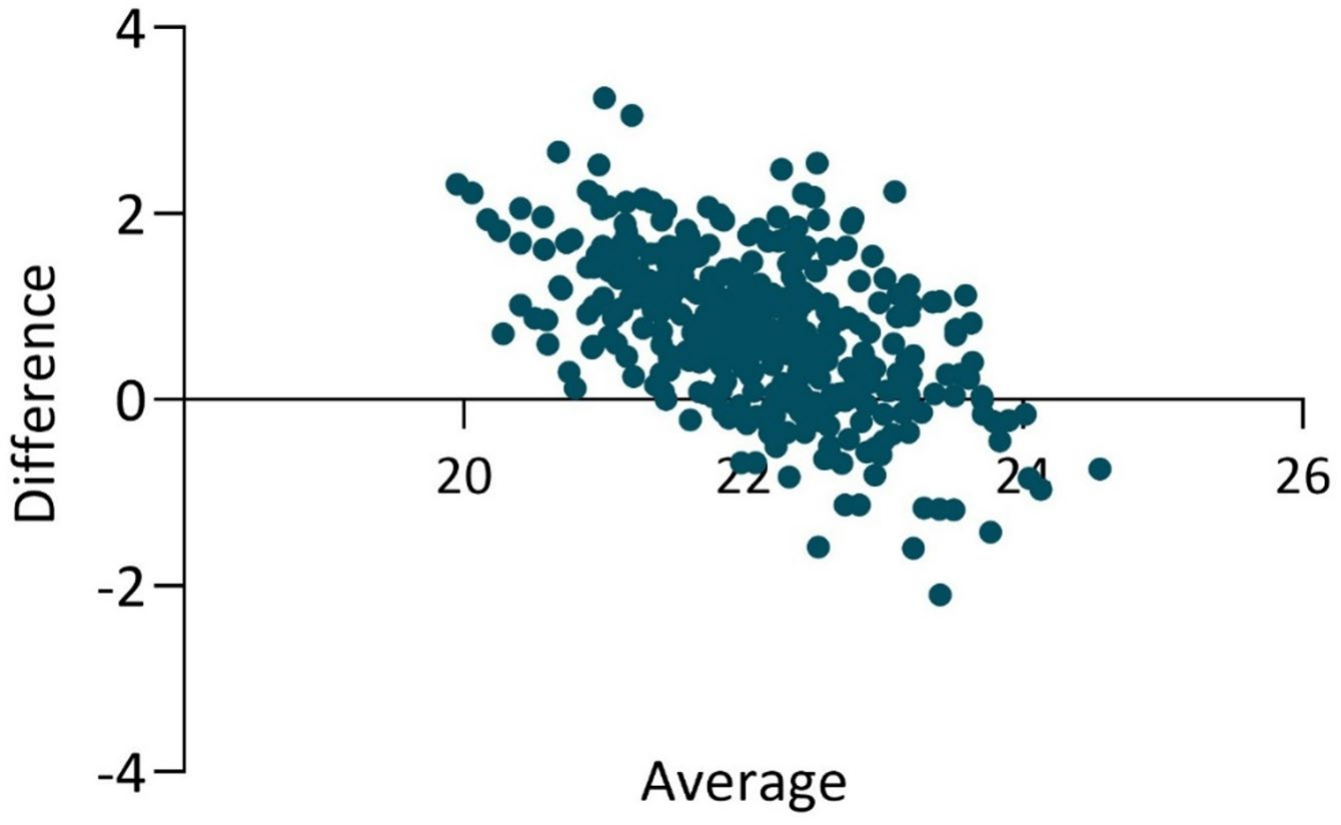
Bland–Altman plot comparing measured values and values predicted using the Tanaka–Johnston equation for maxillary teeth.

**Fig. 4:**
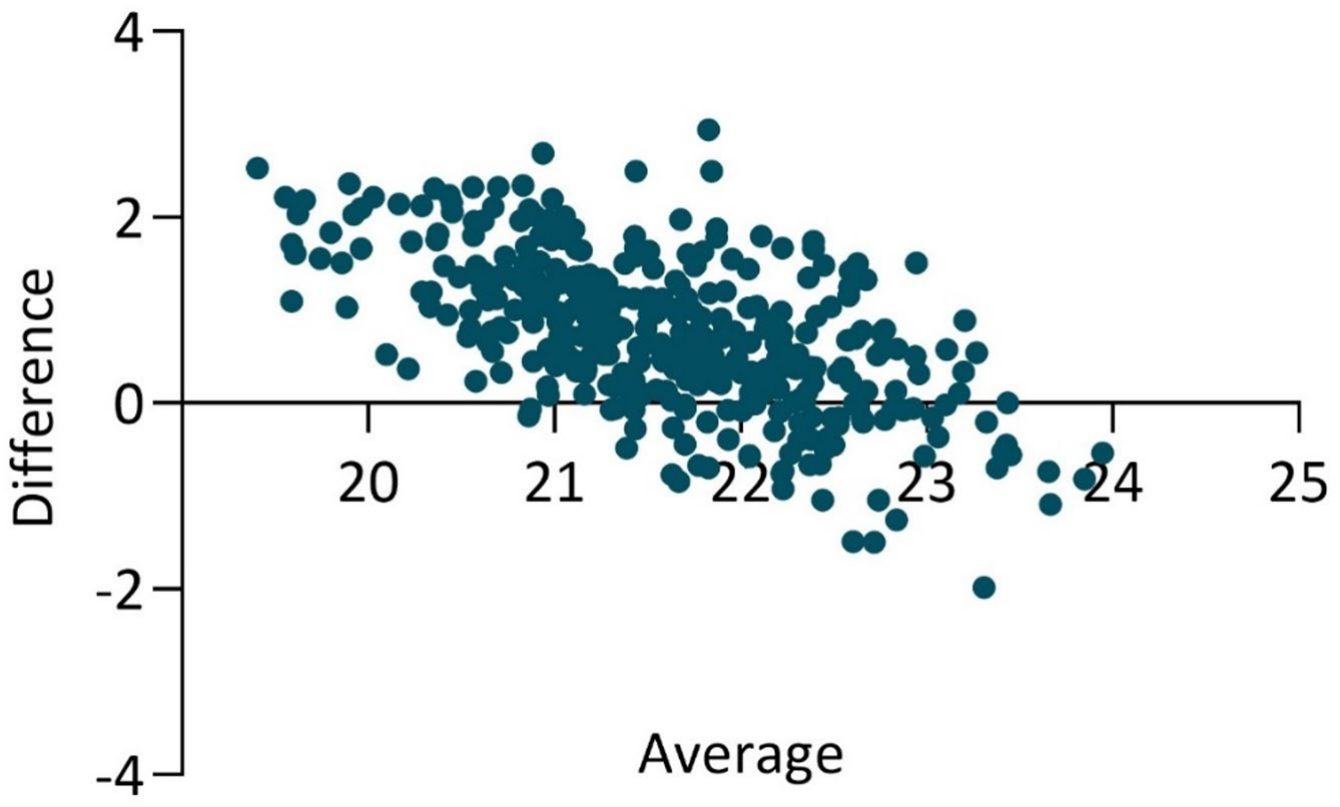
Bland–Altman plot comparing measured values and values predicted using the Tanaka–Johnston equation for mandibular teeth.

Normality testing indicated deviations from normality for the measured values in two tests (Anderson–Darling and Shapiro–Wilk), whereas Tanaka–Johnston predicted values met the normality assumptions. Therefore, both a two-tailed paired t-test and the Wilcoxon signed-rank test were used. For both arches, Tanaka–Johnston predictions differed significantly from the measured widths of canines and premolars (*P* < 0.001). Bland–Altman analysis (differences defined as measured − predicted) showed a systematic negative bias and wide limits of agreement, indicating poor agreement between measured values and Tanaka–Johnston predictions (Table 1; Figs. 3 and 4).

**Table 1:** Bland–Altman analysis of agreement between measured and Tanaka–Johnston predicted widths for maxillary and mandibular teeth.

	Maxillary teeth	Mandibular teeth
Bias	− 0.7712	− 0.7460
SD of differences	0.8160	0.8156
95% Limits of Agreement		
From	− 2.371	− 2.345
To	0.8282	0.8526

### Derivation of new equations

**Fig. 5:**
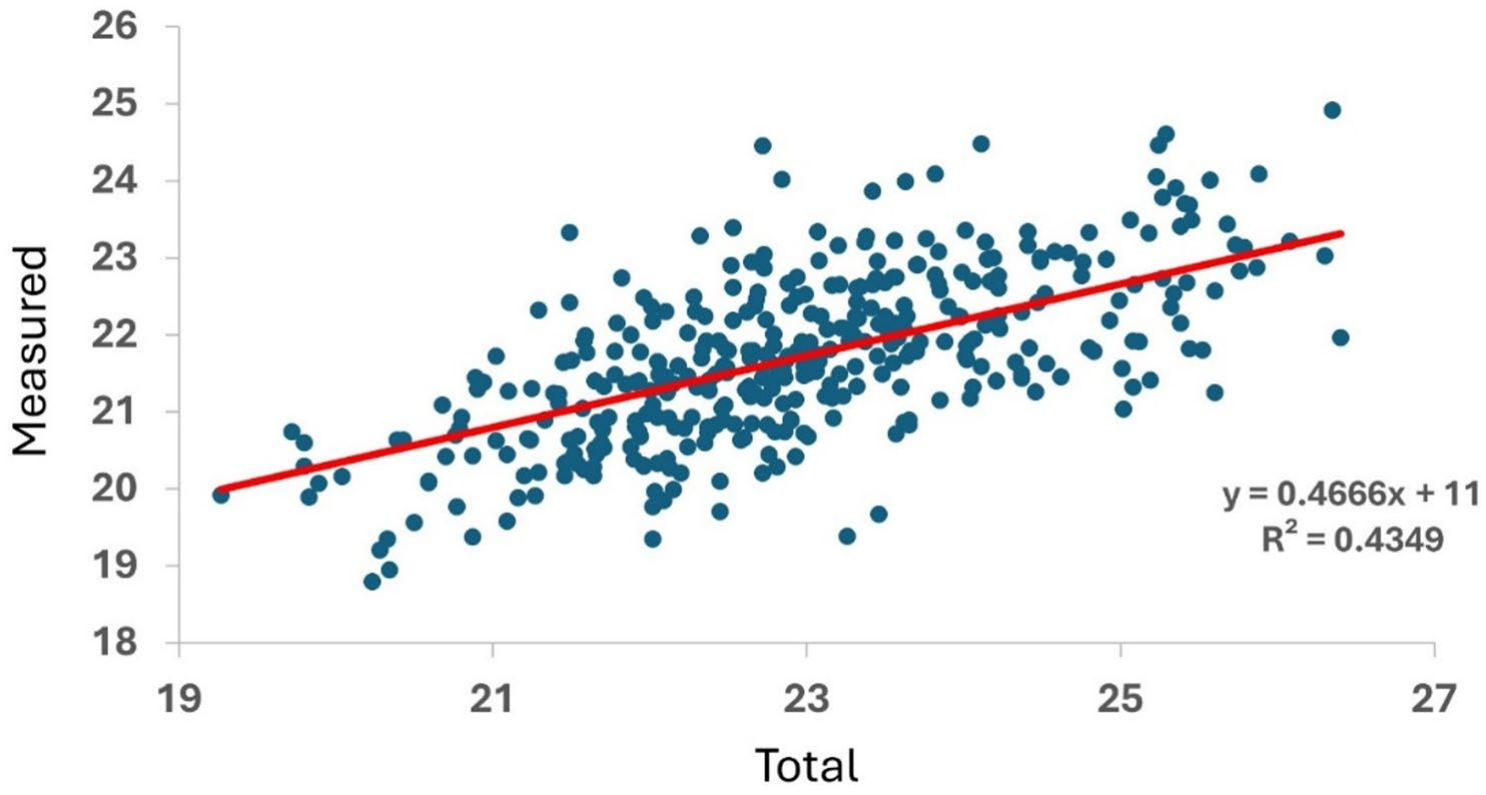
Scatter plot of mandibular incisor widths (total) versus measured maxillary canine–premolars sum, with Tanaka–Johnston predicted values overlaid.

The relationship between mandibular incisor widths and measured canine–premolar sums is illustrated in Fig. 5, which also shows the relative position of Tanaka–Johnston predictions.

New regression equations were derived using mandibular incisor widths as predictors. For models based on the combined mandibular incisor width (“total”), paired t-tests showed no significant difference (*P* > 0.05) between measured and predicted values for either arch (Table 2), and Bland–Altman analysis demonstrated markedly smaller mean bias and narrower limits of agreement compared with Tanaka–Johnston predictions (Table 3).

**Table 2:** Paired t-test comparing measured and predicted widths using the derived equation. (maxillary and mandibular teeth, total mandibular incisor predictor).

Table Analysed	Total maxillary	Total mandibular
Column B	Measured	Measured
vs	vs	vs
Column A	Predicted values	Predicted values
*P* value (*P* < 0.05)	0.497	0.587
t, df	t = 0.6790, df = 378	t = 0.5424, df = 378
Number of pairs	379	379
Mean of differences (B–A)	0.03485	0.02565
SD of differences	0.9992	0.9206
SEM of differences	0.05132	0.04729
95% confidence interval	− 0.06607 to 0.1358	− 0.06733 to 0.1186
R-squared (partial eta squared)	0.001218	0.0007778
Correlation coefficient (r)	0.6594	0.7127
*P*-value (*P* < 0.05)	< 0.001	< 0.001
*P*-value summary	****	****
Was statistically significant difference?	Yes	Yes

**Table 3:** Bland–Altman analysis of agreement between measured and predicted widths using the derived equation (maxillary and mandibular teeth, variable total).

	Maxillary teeth	Mandibular teeth
Bias	− 0.03485	− 0.02565
SD of differences	0.9992	0.9206
95% Limits of Agreement		
From	− 1.993	− 1.830
To	1.924	1.779

Equations derived using mandibular central or lateral incisors showed similar patterns, with no evidence of systematic mean error and comparable agreement. Because these regressions were derived on the full dataset and therefore represent apparent fit, predictive performance was assessed using repeated 70/20/10 training/validation/test splits.

Across repeated runs at a ± 1.0 mm clinical tolerance, linear regression support vector regression (SVR) achieved higher average pass rates than the manually derived equation.

The resulting single-variable, zero-intercept equations were:

$$\begin{gathered} {\mathrm{Predicted}}\;{\text{ combined}}\;{\text{ mesiodistal}}\;{\text{ width}}\;{\text{ of}}\;{\text{ maxillary}}\;{\text{ canine}}\;{\text{ and}}\;{\text{ premolars }}\;{\mathrm{per}}{\mkern 1mu} {\text{ quadrant}}{\mkern 1mu} \hfill \\ \quad \quad \quad = \;{\mkern 1mu} 0.9445 \times \left( {{\mathrm{sum}}{\mkern 1mu} {\text{ of }}{\mkern 1mu} {\mathrm{mandibular}}{\mkern 1mu} {\text{ incisor}}{\mkern 1mu} {\text{ widths}}} \right) \hfill \\ \end{gathered}$$

$$\begin{gathered} {\mathrm{Predicted}}{\mkern 1mu} {\text{ combined}}{\mkern 1mu} {\text{ mesiodistal}}{\mkern 1mu} {\text{ width}}{\mkern 1mu} {\text{ of }}{\mkern 1mu} {\text{mandibular }}{\mkern 1mu} {\text{canine }}{\mkern 1mu} {\text{and }}{\mkern 1mu} {\mathrm{premolars}}{\mkern 1mu} {\text{ per }}{\mkern 1mu} {\mathrm{quadrant}} \hfill \\ \quad \quad \quad = {\mkern 1mu} 0.9242{\mkern 1mu} \times {\mkern 1mu} \left( {{\mathrm{sum}}{\mkern 1mu} {\text{ of}}{\mkern 1mu} {\text{ mandibular}}{\mkern 1mu} {\text{ incisor}}{\mkern 1mu} {\text{ widths}}} \right) \hfill \\ \end{gathered}$$

Across repeated runs, the best-performing linear models included an intercept, with average coefficients corresponding to:

$$\begin{gathered} {\text{Predicted }}\,{\text{combined }}\,{\text{mesiodistal }}\,{\text{width }}\,{\text{of }}\,{\mathrm{maxillary}}\,{\text{ canine }}\,{\mathrm{and}}\,{\text{ premolars}}\,{\text{ per}}\,{\text{ quadrant }} \hfill \\ \quad \quad \quad= \, \,{9}.{7 }\, + \, \,0.{52}\, \, \times \, \, \left( {{\mathrm{sum}}\,{\text{ of }}\,{\mathrm{mandibular}}\,{\text{ incisor }}\,{\mathrm{widths}}} \right) \hfill \\ \end{gathered}$$

$$\begin{gathered} {\mathrm{Predicted}}\,{\text{ combined }}\,{\mathrm{mesiodistal}}\,{\text{ width}}\,{\text{ of}}\,{\text{ mandibular }}\,{\mathrm{canine}}\,{\text{ and }}\,{\mathrm{premolars}}\,{\text{ per }}\,{\mathrm{quadrant}}\, \hfill \\ \quad \quad \quad= {7}.{6} + 0.{55} \times \left( {{\mathrm{sum}}\,{\text{ of}}\,{\text{ mandibular}}\,{\text{ incisor }}\,{\mathrm{widths}}} \right) \hfill \\ \end{gathered}$$

### Machine learning and regression model performance

Model performance was evaluated using repeated 70/20/10 training, validation, and test splits over 50 iterations to enhance robustness and minimize overfitting. Predictive accuracy was assessed using pass rate (± 1 mm tolerance), mean absolute error (MAE), root mean square error (RMSE), and coefficient of determination (R^2^), with average results presented in Table 4. Performance metrics are reported as mean ± standard deviation across repeated iterations.

**Table 4:** Average pass rate (± 1 mm), mean absolute error (MAE), root mean square error (RMSE), and coefficient of determination (R^2^) for regression models using a single predictor across 50 repeated runs (reported as mean ± standard deviation).

Tooth	Method	Runs	Pass rate (%)	MAE (mm)	RMSE (mm)	R^2^
Maxillary	Linear_WI	50	79 ± 7.33	0.65 ± 0.08	0.83 ± 0.10	0.41 ± 0.14
Maxillary	Linear_ZI	50	69 ± 7.10	0.78 ± 0.11	1.00 ± 0.13	0.13 ± 0.31
Maxillary	SVR (Linear)	50	79 ± 7.45	0.65 ± 0.08	0.83 ± 0.10	0.41 ± 0.15
Maxillary	ANN	50	76 ± 6.72	0.69 ± 0.09	0.89 ± 0.13	0.32 ± 0.24
Mandibular	Linear_WI	50	77 ± 7.23	0.65 ± 0.08	0.81 ± 0.08	0.49 ± 0.09
Mandibular	Linear_ZI	50	73 ± 6.51	0.71 ± 0.09	0.91 ± 0.11	0.36 ± 0.17
Mandibular	SVR (Linear)	50	77 ± 7.61	0.65 ± 0.07	0.81 ± 0.08	0.50 ± 0.09
Mandibular	ANN	50	75 ± 7.38	0.68 ± 0.08	0.86 ± 0.10	0.44 ± 0.13

Linear WI: Linear regression with intercept; Linear ZI: Linear regression without intercept; SVR: Support vector regression (linear kernel); ANN: Artificial neural network.

Across repeated runs, linear regression with intercept (Linear_WI) and support vector regression (SVR) demonstrated the highest and most consistent performance for both maxillary and mandibular predictions, achieving mean pass rates of up to 80%, with lower error values (MAE ≈ 0.65–0.66 mm; RMSE ≈ 0.81–0.82 mm) and moderate R^2^ values. Artificial neural network models showed comparable but slightly lower performance, whereas linear regression without an intercept (Linear_ZI) consistently demonstrated lower accuracy and higher prediction error.

Table 5 presents the best-performing models, highlighting those achieving the highest pass rates and lowest MAE values across iterations, together with their corresponding regression coefficients. These findings indicate that optimized linear regression models achieved high predictive accuracy with clinically acceptable error margins.

**Table 5:** Best-performing regression models using a single predictor across 50 repeated runs (± 1 mm tolerance) (a) Models achieving the highest pass rates (b) Models achieving the lowest MAE.

	Arch	Method	Run	Pass rate (%)	MAE (mm)	RMSE (mm)	R^2^	Intercept	Coefficient
a	Maxillary	Linear_WI	19	97	0.59	0.71	0.57	10.00	0.50
	Mandibular	Linear_WI	9	89	0.57	0.76	0.50	7.63	0.59
b	Maxillary	Linear_WI	33	84	0.51	0.65	0.52	9.98	0.51
	Mandibular	Linear_ZI	39	81	0.45	0.63	0.68	0.00	0.92

Linear_WI: Linear regression with intercept; Linear_ZI: Linear regression without intercept.

MAE: mean absolute error; RMSE: root mean square error; R^2^: coefficient of determination.

A comparative summary of all methods is provided in Table 6. Machine-learning models achieved higher overall performance (average pass rate: 79%) than the Tanaka–Johnston method (59%) and the newly derived regression equation (70%), with lower prediction errors and improved goodness-of-fit. Notably, the best-performing machine-learning models achieved pass rates of up to 97%. At the same time, the lowest prediction error reached an MAE of 0.45 mm (RMSE: 0.63 mm), indicating enhanced predictive accuracy within clinically acceptable limits.

**Table 6:** Comparison of predictive performance across all methods evaluated in this study.

Model	Pass rate (%)	MAE (mm)	RMSE (mm)	R^2^
Tanaka-Johnston	59	0.92	1.11	0.05
New regression equation (this study)	70	0.74	0.96	0.29
Machine learning (average)	79	0.66	0.82	0.45
Machine learning (best pass rate)	97	0.59	0.71	0.57
Machine learning (lowest MAE)	81	0.45	0.63	0.68

## Discussion

Population-specific calibration and machine learning-assisted prediction models for MDSA have increased recently, reflecting a shift from universal regression equations toward locally validated methods^13,14^. The present study assessed the validity of the Tanaka–Johnston mixed dentition analysis in an Emirati population and developed population-specific regression and machine learning models to improve the prediction of unerupted canines and premolars widths. The results revealed apparent limitations with traditional prediction equations when applied across populations and emphasize the importance of locally calibrated models supported by modern analytical methods.

Given significant disagreement between Tanaka–Johnston predictions and the measured values, with a systematic negative bias and wide limits of agreement, the null hypothesis was rejected. These findings are consistent with considerable evidence suggesting that MDSA derived from Northern European populations may not be universally appropriate. Ethnic and sex-related variation in mesiodistal tooth widths has been well reported and can significantly affect the precision of regression-based predictions^10^. Comparable discrepancies have been reported across multiple populations. Studies in Saudi and Syrian cohorts showed consistent overestimation of canine and premolar widths using Tanaka–Johnston equations, often exceeding clinically accepted thresholds of approximately 1 mm^11,24,25^. Similar limitations have been reported in Northeast Han Chinese and Nepalese populations, where the development of population-specific regression models significantly improved predictive performance^26,27^. Systematic reviews also indicate that the Moyers and Tanaka–Johnston analyses show varying accuracy across ethnic groups and require local validation before clinical application^28^. In addition to the current findings, these studies reinforce the concept that universal application of classical regression equations may lead to clinically significant prediction errors.

Compared with Tanaka–Johnston predictions, the new regression equations derived from the sum of mandibular incisor widths showed significantly better agreement with the measured combined width of canine and premolars per quadrant. The absence of significant mean error and the reduction in Bland–Altman bias indicate improved calibration to the Emirati dentition.

These results are largely consistent with regional investigations. Burhan (2014) and Al-Fraidi (2024)^11,24^ reported that population-specific regression equations substantially reduced prediction error compared with traditional methods in Syrian and Saudi samples. Similarly, studies conducted on Nepalese and Indian populations have shown that recently derived regression models achieved stronger correlations and narrower error ranges than Moyers or Tanaka–Johnston analyses^26,29^. The strong association observed between the sum of mandibular incisor widths and the combined width of canine and premolars per quadrant supports longstanding orthodontic principles that identify lower incisors as reliable early predictors due to their early eruption and stable statistical relationship with posterior tooth width^11^.

In various population groups, prediction errors exceeding approximately ± 1 mm have been associated with clinically relevant differences in MDSA. Therefore, the reduced bias and narrower agreement limits achieved by the current equations indicated an improvement in clinical validity compared to imported standards.

Machine-learning approaches improved predictive performance but did not fundamentally alter the underlying biological relationships in mixed-dentition space analysis. Linear regression with intercept and support vector regression consistently revealed the highest and most stable performance across repeated runs, with higher pass rates, lower error values (MAE and RMSE), and moderate goodness-of-fit (R^2^). In contrast, artificial neural network models did not reveal a consistent advantage.

The dataset’s underlying characteristics can clarify why machine learning and deep learning models do not perform well. The predictive relationship was mainly linear and based on a single variable, limiting the potential benefit of more complex nonlinear algorithms. In such settings, classical linear regression is usually satisfactory, while increased model complexity may present additional variance without enhancing predictive accuracy.

These findings demonstrate that the relationship between the sum of mandibular incisor widths and the combined width of canines and premolars per quadrant remains predominantly linear, and increasing algorithmic complexity does not necessarily translate into improved clinical performance.

This observation is consistent with emerging research on artificial intelligence in orthodontics. Moghimi et al.^14^ reported enhanced predictive accuracy with a hybrid genetic algorithm–ANN system, while Camcı and Salmanpour^13^ showed that deep learning models may outperform traditional methods such as Moyers analysis. However, the present findings extend this evidence by showing that well-calibrated linear models can achieve comparable predictive accuracy, as reflected by similar pass rates and error metrics, while maintaining greater interpretability. Comparable conclusions have been documented in methodological studies, where simpler regression models performed reliably when properly validated^7^.

The comparable performance of single-predictor models further highlights the clinical relevance of mandibular incisor width as a key determinant of posterior tooth size. Rather than replacing established orthodontic principles, machine-learning approaches in the current study primarily enhanced coefficient estimation and improved prediction stability across repeated iterations.

The observed differences should be interpreted in the context of clinical thresholds, where prediction errors within ± 1 mm are generally considered acceptable for orthodontic decision-making.

The variation in tooth size within each population group is likely to clarify the improved performance of the newly derived equations. A previous study showed strong correlations among tooth width, sex, and ethnicity, with males typically exhibiting greater mesiodistal widths, and ethnic differences influencing regression coefficients in MDSA^10^. Studies conducted on groups of Chinese, Nepalese, Saudi, and Syrian cohorts have confirmed the need for region-specific predictive models^11,25–27^.

The Emirati population represents a distinct demographic group within the Middle East, and the current results extend the existing literature by confirming that modified regression equations calibrated to local populations provide improved clinical reliability compared to traditional standards.

The present results should also be interpreted in the context of the rapidly expanding role of artificial intelligence in orthodontics. Recent studies have shown that machine-learning and deep-learning approaches can improve diagnostic accuracy, treatment planning, and the prediction of clinical outcomes by identifying complex patterns in large datasets^30^.

Artificial intelligence has been applied across multiple disciplines in orthodontics, including cephalometric analysis, landmark detection, growth assessment, and extraction decision-making, with reported progress in efficiency and reduction in operator variability^31^.

However, despite these advances, current evidence suggests that the performance of machine-learning models is highly dependent on dataset characteristics, model complexity, and clinical context. In structured, biologically linear relationships, such as those between mandibular incisor widths and canine–premolar dimensions, simpler, interpretable regression models may achieve comparable predictive performance^32^.

The findings of the present study support this perspective, indicating that machine-learning approaches primarily enhanced prediction stability rather than fundamentally outperforming well-calibrated linear regression models. This emphasizes the importance of balancing model complexity with clinical interpretability when incorporating artificial intelligence into orthodontic practice.

### Clinical implications

From a clinical perspective, the systematic bias observed in Tanaka–Johnston predictions underscores the potential for inaccurate mixed-dentition space analysis when classical equations are applied without population-specific validation. Inaccurate estimation of canine and premolar widths may directly affect treatment planning decisions, including space supervision, serial extraction, and interceptive orthodontic mechanics. Overestimation of tooth size may lead to unnecessary extractions or space-gaining procedures, whereas underestimation may increase the risk of crowding, delayed eruption, and canine impaction.

In contrast, the newly developed regression equations showed improved agreement with measured values, supporting their use as population-specific tools for more accurate clinical prediction. Improved prediction accuracy enables more reliable estimates of space requirements, facilitating early and suitable intervention. Notably, the results indicate that machine-learning–assisted approaches primarily improve coefficient optimization rather than replace established orthodontic principles, keeping the simplicity, transparency, and clinical applicability required for routine use.

### Strengths and limitations

A major strength of this study is the development of prediction models and their internal validation using a relatively large sample of Emiratis. This population is still underrepresented in orthodontic research. The integration of repeated training, validation, and testing with Bland–Altman agreement analysis allowed for evaluation of both statistical performance and clinical applicability.

Several limitations should be considered when interpreting the findings. The developed models are population-specific, as they were derived from an Emirati sample, and their generalizability to other populations may be limited. Although internal validation was performed using repeated data partitioning to enhance model robustness, external validation in independent cohorts was not conducted and remains necessary.

Although data were collected from multiple centers, all centers operated within the same healthcare system and followed standardized clinical protocols, which likely minimized inter-center variability. However, as a retrospective study, potential selection bias and residual heterogeneity cannot be entirely excluded.

The biological variables, such as sex and age, were not incorporated into the predictive models. While mesiodistal tooth dimensions are generally stable after eruption, sexual dimorphism and age-related variability may influence prediction accuracy. The inclusion of these variables would require larger datasets to allow adequately powered stratified or multivariable modeling without increasing the risk of overfitting. Future studies should therefore evaluate their contribution to predictive performance.

The sample size was determined based on a clinically relevant precision approach rather than model-specific power analysis for regression and machine-learning techniques. Although the relatively large sample size and repeated validation strategy were considered sufficient to support model stability, future studies should employ formal model-based sample size calculations.

The absence of formal multiple comparison correction may increase the risk of type I error and should be considered when interpreting the statistical results.

Although model performance variability is now reported as mean ± standard deviation across repeated data splits, uncertainty estimates for regression coefficients were not provided, as the study focused on predictive performance rather than inferential modeling. Future studies should incorporate such analyses to further enhance model interpretability.

## Conclusions

The Tanaka–Johnston method demonstrated limited validity in the Emirati population, with significant differences and reduced clinical reliability. Population-specific regression equations derived from mandibular incisor widths significantly enhanced predictive accuracy, as reflected by higher pass rates and lower prediction error, while maintaining the simplicity required for clinical application. Machine-learning–assisted modeling improved prediction stability but did not provide a consistent advantage over well-calibrated linear regression models, supporting the primarily linear nature of tooth-size relationships.

These findings support the use of locally derived, interpretable prediction equations for mixed-dentition space analysis, enabling more accurate estimation of space requirements and more informed clinical decision-making in orthodontic treatment planning, including space management and extraction decisions.

## Data Availability

The datasets generated and/or analysed during the current study are available from the corresponding author on reasonable request.

## Abbreviations

MDSA: Mixed dentition space analysis
AI: Artificial intelligence
DL: Deep learning
ML: Machine learning
ICCs: Intraclass correlation coefficients
SVR: Support vector regression
ANN: Artificial neural networks
MAE: Mean Absolute Error
RMSE: Root Mean Square Error
(R^2^): Coefficient of Determination
